# Synergistic remediation of oil-water mixtures: integration of nanoparticles and oil-degrading bacteria in a novel approach

**DOI:** 10.3389/fmicb.2025.1590636

**Published:** 2025-07-02

**Authors:** Marwa E. El-Sesy, Sabah S. Ibrahim, Fan Yang, Abeer Hegazy

**Affiliations:** ^1^Central Lab for Environmental Quality Monitoring, National Water Research Center, Cairo, Egypt; ^2^Institute of Vegetable, Henan Academy of Agricultural Sciences, Zhengzhou, China

**Keywords:** bioremediation, nano bioremediation, *Artemisia* leaves, oil-degrading microorganism, water quality

## Abstract

Oil Spillage is considered one of the environmental disasters due to the release of hydrocarbons into the aquatic environment, causing destructive effects. Usually, it occurs accidentally or intentionally, mostly resulting from human activities. Effective degradation of these spills is vital to preserving the aquatic ecosystem. Recently, combining state-of-the-art nanotechnology with degrading bacteria is an area of great interest and opens new paths for addressing oil/water pollution issues. In this context, the current study assesses the synergetic effect of a hybrid of green-synthesized magnetite nanoparticles and oil-degrading bacteria on oil spill removal *in vitro* conditions. Bacterial isolates were isolated from water samples collected from Sharkia, Qalyubia, and Minufiya Governorates in Egypt. Effective oil-degrading strains were identified by 16S rRNA gene sequencing. Magnetite nanoparticles (AL-MNPs) were synthesized from Artemisia leaf extract through green synthesis and characterized using UV-Vis spectroscopy, TEM, EDX, and FTIR. Bacterial growth was compared with the effect of AL-MNPs at various concentrations and exposure durations. Results indicated that the interaction between AL-MNPs and bacteria greatly enhanced oil degradation. With optimal conditions (35°C, pH 7, 300 mg/l oil, and 0.04 g AL-MNPs), almost 90% of oil was degraded after 3 days. AL-MNPs alone degraded about 59%, and bacteria alone degraded 72–80% in the same period. GC-MS analysis verified that the system degraded almost 50% lower-chain alkanes (C9–C22) and 30% higher-chain alkanes (C23–C26). Furthermore, the recyclability of the AL-MNPs was also explored, with slight loss of removal efficiency upon repeated usage, but with superior performance. This demonstrates the long-term capability of the AL-MNPs-bacteria system. Correspondingly, the eco-friendly synthesis of AL-MNPs using plant extracts reinforces their role as a sustainable and cost-effective alternative to oil spill degradation techniques. The most astounding result, the synergizing effect between oil-degrading bacteria, environmentally isolated, and AL-MNPs. This not only enhances the degradation process but also contributes to a greener, more integrated remediation strategy that aligns with circular economy and environmental protection goals.

## Graphical Abstract

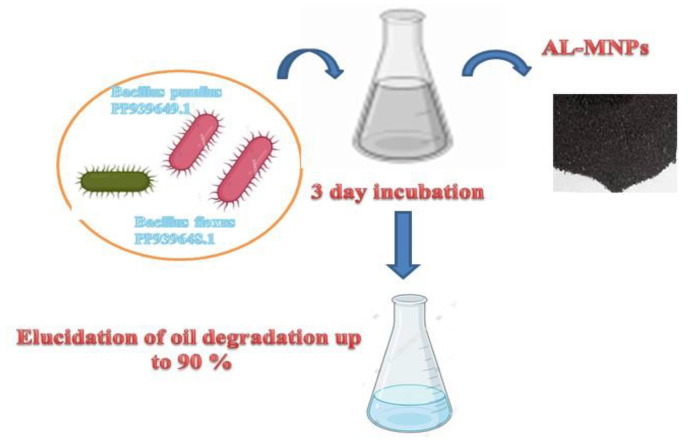

## 1 Introduction

Petroleum hydrocarbon compounds such as n-alkanes, cycloalkanes, and aromatic hydrocarbons are among the most harmful environmental pollutants released into water bodies (Ahmed et al., 2024). They are released into aquatic environments through various human activities such as major petroleum exploration, transportation, refining, storage, and spilling (Kaur et al., 2023). As hydrophobic and persistent petroleum hydrocarbons, they will bioaccumulate in aquatic environments and cause massive ecological disruption (Gikonyo et al., 2022; Varjani et al., 2020). If they are released in water, the compounds may form surface films, which inhibit the exchange of oxygen, block the penetration of sunlight, and have a direct impact on aquatic flora and fauna. Organic toxic pollutants, especially in inland or slow waters, lead to oxygen depletion, loss of habitats, and biodiversity impacts in the long term (Marghoob et al., 2022). Freshwater and marine ecosystems are most vulnerable to hydrocarbon contamination. Total petroleum hydrocarbons (TPHs) are known to cause dramatic dangers to aquatic life via physiologic, reproductive, as well as behavioral abnormalities in fishes and invertebrates (Macoustra et al., 2021). In addition to the damaging effects they inflict on biodiversity, such pollutants bring huge challenges to drinking water treatment systems (Zainab et al., 2023). The majority of petroleum hydrocarbons are recalcitrant to conventional treatment technologies and can persist in treated effluents, which is harmful to human and animal health (Liang and Esmaeili, 2021).

Several remediation technologies have been developed to treat petroleum-contaminated water, including pump-and-treat systems, chemical oxidation, air sparging, membrane filtration, activated carbon adsorption, and flotation (Kshirsagar et al., 2020; Kumar et al., 2022). While such technologies can reduce levels of pollutants, they are invariably high in running costs, complex in infrastructure, and in the provision of means that do not completely break down target pollutants (Feng and Zhang, 2023). Moreover, physical and chemical treatments are mostly nothing but transferring the contaminants from one phase to another, e.g., from aqueous to solid, and require additional treatment or disposal and suffer from secondary pollution problems (Amran et al., 2022). On the other hand, bioremediation has become a potential substitute for its eco-friendly and low-cost nature. The technique exploits the metabolic process of microbes in breaking down and detoxifying petroleum hydrocarbons from water pollution (Chakravarty et al., 2024). Microorganisms like *Pseudomonas*, *Bacillus*, and *Acinetobacter* species have been extensively researched for their capacity to metabolize hydrocarbons into simpler less toxic substances (Kaewlaoyoong et al., 2020). Bioremediation methods typically encompass bioaugmentation (addition of hydrocarbon-degrading microbial populations) and biostimulation (activation of microbial action through the optimization of environmental and nutritional parameters). Nutrient supplementation, particularly for nitrogen and phosphorus, is pivotal for microbial development and enzymatic function in aquatic systems (Sattar et al., 2022).

However, despite its advantages, bioremediation alone has its limitations. One of the primary constraints is the poor bioavailability of hydrophobic hydrocarbons in aquatic environments, especially with high molecular weight and low solubility (Oba et al., 2022). These hydrocarbons may readily form micelles or be adsorbed onto particulate matter and reduce their exposure to microbial enzymes. In others, the indigenous flora of hydrocarbon-degrading bacteria may not be sufficient or maybe in a dormant state under prevailing environmental conditions, leading to retardation of the rate of degradation (Mohanta et al., 2024). Moreover, the majority of bacteria possess optimal pH, temperature, and oxygen levels required for effective function—levels habitually disrupted in contaminated systems (Chaudhary et al., 2023).

As a response to these challenges and in order to enhance hydrocarbon degradation in water, researchers have increasingly focused on nanotechnology-based approaches (Kim and Cha, 2021). Nanoparticles (NPs) offer a novel and exciting avenue to further optimize the effectiveness of bioremediation through enhanced pollutant accessibility and microbial activity (Sowndarya et al., 2024). Due to their nano-scale dimensions and high surface area-to-volume ratio, NPs have the ability to interact with contaminants as well as microbial cells more effectively (Thangadurai et al., 2021; Sharma et al., 2020). They can be used as adsorbents, catalysts, or carriers to facilitate increased mass transfer as well as improve the surface accessibility of hydrocarbons to microbes. Experiments have shown that certain metal oxide nanoparticles, such as magnetite nanoparticles (Fe_3_O_4_), zinc oxide (ZnO), and titanium dioxide (TiO_2_), can enhance the microbial degradation of hydrocarbons in aqueous systems (Yadav et al., 2022). Besides improving the dispersion of hydrophobic chemicals, these NPs improve microbial colonization and metabolism. For example, magnetite nanoparticles can decrease interfacial tension and oil viscosity, thereby making it easier for microorganisms to break it down (El-Hoshoudy et al., 2021). Nanoparticles can also ensure good redox conditions and provide trace elements required for microbial growth (Torres-Rivero et al., 2021).

Furthermore, green nanoparticle synthesis—by plant extracts or biological processes—is rapidly gaining acceptance due to ease of operation, low cost, and low environmental impact (Choudhary et al., 2022). More particularly, magnetite nanoparticles from plant extracts have exhibited high potential in environmental remediation (Ali Redha, 2020). Not only are the biologically synthesized nanoparticles (AL-MNPs) environmentally safe, but also they are rich in surface-functional groups, enabling adsorption and catalytic degradation of organic contaminants (Gupta et al., 2023). Green magnetite nanoparticles synthesized also offer the advantages of ease of handling and recovery after post-treatment. They can be easily recovered from treated water through the use of external magnets due to their magnetic properties, reducing the likelihood of accumulation in ecosystems. They can be reused after regeneration. Combined with microbial treatment, such nanoparticles can significantly increase the overall effectiveness of hydrocarbon degradation even when environmental conditions are not optimal (Kumar et al., 2022).

Combined treatment of AL-MNPs with hydrocarbon-degrading bacteria is a synergistic treatment of oil-contaminated water. The nanoparticles act as promoters by enhancing the dispersion of contaminants and triggering bacterial activity, while microbes are responsible for performing the actual bioremediation task. This nano-bio hybrid system has been found to reduce the degradation time significantly as well as increase the overall efficiency of oil removal. For instance, NPs can attach to the cell walls of bacteria, altering cell permeability and surface charge, which improves the uptake of substrates and hydrolysis by enzymes (Srivastava et al., 2021). Moreover, nanoparticles can serve as carriers for enzymes or nutrients, further improving microbial performance. There are several studies that have also debated the application of nanoparticles as sensor systems for monitoring remediation process. Microbial activity, intermediate products, and real-time remediation efficiency feedback can be measured by these sensors (Koul et al., 2021). Incorporating such technology in remediation methods not only makes the process more efficient but also supports data-based environmental management strategies (Gupta et al., 2023).

In the current study, we focus on the use of biosynthesized magnetite nanoparticles through *Artemisia* leaf extract (AL-MNPs) in combination with native hydrocarbon-degrading bacteria that were isolated from water samples at the ship settlement station in Alkanater City, Qalyubia Governorate, Egypt. This is to evaluate the enhanced degradation of petroleum-based pollutants—specifically vegetable oil and diesel—from contaminated water through synergistic interaction between AL-MNPs and microbial agents. The novelty of this study is the application of native microbial consortia together with green nanoparticles for the resolution of one of the major environmental issues. This method eliminates secondary pollution, decreases treatment duration, and is a sustainable strategy for large-scale water detoxification in comparison with conventional remediation technologies. This research contributes to the developing field of integrated environmental remediation technologies by combining microbial bioremediation and green nanotechnology and opens the door for future studies attempting to mitigate the impacts of petroleum pollution in aquatic ecosystems.

## 2 Materials and methods

### 2.1 Chemicals, solutions, and materials

Artemisia leaves were obtained from the Agricultural Research Center (ARC), Cairo, Egypt. All chemicals, including materials used in the nanoparticle synthesis process, were of the highest purity of analytical grade and sourced from Al-Gomhorya Company for several chemicals in Egypt. The substrates for preparing minimal salt broth were acquired from Merck, Germany. Regarding the source of oil, the engine oil was sourced from a gasoline station in Egypt, while sunflower as a vegetable oil, representing non-petroleum oils, was acquired from the nearby marketplace in Cairo, Egypt.

### 2.2 Sample collection

Twenty-one water samples were collected and analyzed according to the guidelines outlined by the American Public Health Association (APHA) (2017). To isolate oil-degrading bacteria, the samples were chosen from three main sites as deposited in Table 1; the first location was Ismalia canal at Belbies city, Al-Sharqiagovernorate (S1), the second was River Nile at the ship settlement station, Al Qanatir Al Khayriyyah city, Al-Qalyubia governorate (S2), and the third one was El Khadrawia drain, Menofia Governorate (S3). Collected samples were transported in sterile bottles and stored at 4°C for further assessments. Water quality parameters were assessed, including physical, chemical and bacterial parameters.

**TABLE 1:** Locations and characterization of water samples from various locations.

Location name	Location latitudes	Number of samples and codes	Characterized water
Ismalia canal at Belbies city, Al-Sharqia governorate (S1)	30°25′05.0′′N 31±34′34.0′′E	S1–1, S1–2, S1–3, S1–4, S1–5, S1–7	Clear raw water
Al Qanatir Al Khayriyyah city, Al-Qalyubia governorate (S2)	30°09′40.6′′N 31±08′05.8′′E	S2–1, S2–2, S2–3, S2–4, S2–5, S2–6, S2–7	Water contaminated with oil due to occasional spills from ship maintenance activities
El Khadrawia drain, Menofia Governorate (S3)	30°32′48.4′′N 31±11′27.7′′E	S3–1, S3–2, S3–3, S3–4, S3–5, S3–7	Highly polluted wastewater

### 2.3 Isolation and screening of oil degradation bacteria

Bacteria were isolated from the different samples using the pour plate method No. 9215B (American Public Health Association (APHA), 2017) and maintained at 4°C. Screening of the bacterial isolates was performed by inoculating them individually in 250 mL glass conical flasks containing 50 mL of mineral salts medium (MSM), formulated according to Ewida (2014): 0.6 g Na_2_HPO_4_, 0.2 g KH_2_PO_4_, 4.0 g NaNO_3_, 0.3 g MgSO_4_, 0.01 g CaCl_2_, 0.01 g FeSO_4_ per liter of deionized water, with 0.1% vegetable oil added as the sole carbon and energy source. The cultures were incubated at 30°C on a rotary shaker at 150 rpm for 12 days. Once more, 5 mL of the enriched culture was transferred to fresh medium and incubated, and the process was repeated four times to enrich the cultures as recommended by Ewida and Mohamed (2019). The enriched cultures were inoculated with different vegetable oil concentrations: 0.2, 0.5, 0.7, 1, 2, and 5%, and biochemical tests were then performed on the degraded oil. Residual oil concentrations in the enriched samples were monitored according to Method 5520 B—Partition-Gravimetric Method, as outlined in the Standard Methods for the Examination of Water and Wastewater (American Public Health Association (APHA), 2017). This method involves the extraction step of oil and grease using an organic solvent, followed by gravimetric determination of the residue after solvent evaporation. A visible spectrum spectrophotometer (Hitachi U-2910, Tokyo, Japan) was used to measure optical density (OD) at 600 nm, a commonly used standard for estimating bacterial growth, as it provides a reliable indicator of cell density in liquid cultures. Take a sample of 1 mL from the suspension culture and transfer into a cuvette. Then, measure the OD600 against the sterile broth as the blank.

Measurements were taken before and 10 days after inoculation in a mineral salt medium to monitor the biomass of the cultures. In addition, the ability of bacteria to degrade oil was tested times. Using phenotypic identification techniques, including Gram-staining and culture-based methods, was used for determining the potential isolates with the highest oil degradation.

### 2.4 Molecular identification of biodegrading bacteria using 16S rRNA gene sequencing

One of the genotypic analyses to confirm the best oil-degrading isolates, 16S rRNA gene sequencing analysis, was performed after biochemical and morphological tests.

#### 2.4.1 DNA extraction and PCR amplification

DNA of each bacterium was extracted and purified using the protocol outlined by Sambrook et al. (1989). PCR amplification of bacterial isolates utilized universal primers RS_001101.5F (5′-CCGCGGTAATACAGAGGGTG-3′) and 1492R (5′-ACCGCCCTCTTTGCAGTTAG-3′), as described by Nilsson et al. (2019). The PCR thermal profile consisted of initial denaturation at 94°C for 5 min, followed by 30 cycles of denaturation at 94°C for 1 min, annealing at 55°C for 1 min, extension at 72°C for 2 min, and a final extension at 72°C for 10 min.

#### 2.4.2 Bioinformatics analysis

DNA sequences obtained were subjected to analysis using the Basic Local Alignment Search Tool (BLAST) available on the NCBI database^1^ to identify closely related sequences from GenBank. The CLUSTAL W program was employed for sequence alignment, and phylogenetic relationships were determined using Molecular Evolutionary Genetics Analysis (MEGA 7.0) software (Tamura et al., 2013). A Maximum Likelihood phylogenetic tree was constructed with bootstrap values derived from 1,000 replicate runs, following the approach described by Verma et al. (2015).

This comprehensive analysis facilitated the identification and characterization of the most proficient crude oil-degrading bacteria based on their genetic profile and evolutionary relationships with known bacterial species.

### 2.5 Green synthesis of magnetite nanoparticles from *Artemisia* leaf extracts (AL-MNPs)

**FIGURE 1:**
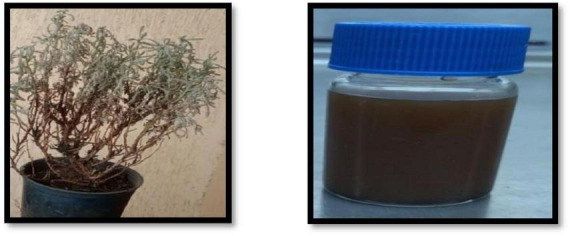
Magnetite biosynthesis was performed utilizing *Artemisia* leaves extract.

Magnetite biosynthesis was carried out using a plant extract, as described by Harikrishnan et al. (2024), This is the first time that *Artemisia* leaves have been used as a plant extract in green biosynthesis, as shown in Figure 1. In order to prepare the plant extract, thirty grams of plant leaves were thoroughly cleansed with deionized water, followed by drying and grinding using a mortar. The resulting powder was immersed in 100.0 mL of deionized water and subjected to continuous agitation (∼ 100 rpm) for 20 min at 75°C to enhance the extraction of bioactive compounds. After natural cooling of the homogenized solution, it was filtered through a Whatman filter paper (diameter 45 mm) to obtain a pure liquid solution (supernatant), which was then stored for subsequent nano-synthesis. Following this, the green synthesis method of Fe_3_O_4_-NP was applied by preparing a solution containing 50.0 mL of FeCl_2_.4H_2_O and FeCl_3_.6H_2_O in a 1:2 M ratio in deionized water, and then stirred at 75.0°C for 20 min. Afterward, the iron mixture solution was combined with 10.0 mL of the supernatant under agitation (∼ 100 rpm) at 65.0°C for 5.0 h. An alkaline solution of 1.0 M NaOH (30 mL) was then gradually added (e.g., 2 mL min^–1^) to the reaction mixture to induce consistent precipitation of nanoparticles (NPs) while maintaining the pH at 11. The temperature was maintained at 85°C throughout the 4-h experiment. Following the synthesis process, the resulting AL-MNPs were magnetically separated from the aqueous medium using a neodymium magnet, washed repeatedly with deionized water, methanol, and ethanol, and then dehydrated for 3 h in an oven at 60°C. Finally, the material was prepared for nano-bioremediation applications.

### 2.6 Characterization techniques of AL-MNPs

In recent years, the characterization of NPs has received significant attention using ultraviolet-visible (Uv-vis), Fourier Transform Infrared Spectroscopy (FTIR), X-ray diffraction (XRD), Energy-dispersive X-ray spectroscopy (EDS) and Field emission scanning electron microscopy (FESEM) techniques (Paturi et al., 2020). Thus, the synthesis confirmation and characterization of AL-MNPs were performed using UV-Vis, Energy-dispersive X-ray spectroscopy (EDX), Transmission electron microscopy (TEM), Scanning electron microscopy (SEM) and Fourier Transform Infrared Spectroscopy (FTIR). Employing UV-Vis spectroscopy in a Hitachi U-2910 Spectrophotometer (Tokyo, Japan), continuous scanning was conducted across 200–800 nm wavelengths to analyze the absorption spectra. TEM, 100 kV, JEM 2100Plus, JEOL Limited, Japan, was used to elucidate particle size, morphology, and particle dispersion for the morphological and structural characterizations. This was followed by SEM at 10 kV by the JSM 8404 of the same JEOL, and magnified images were taken at × 50,000 and × 1,00,000 for capturing the surface features of the samples in detail. EDX was performed in combination with SEM to determine the elemental composition of AL-MNPs, confirming the presence of major elements and the purity of the synthesized nanoparticles. FTIR analysis was carried out using a Perkin-Elmer FTIR 1650 spectrophotometer to study the functional groups and chemical bonds on the surface of the AL-MNPs. The diversity techniques used to characterize the synthesized AL-MNPs provide important information about their synthesis, structure-morphology, and chemical composition, potentially making them highly valuable for further applications.

### 2.7 Experimental setup for bioremediation

#### 2.7.1 Effects of environmental factors on bacterial growth

Equalized bacterial suspensions were used to optimization some of the physic-chemical factor such as temperature, pH, phosphate, and nitrate.

To investigate the impact of environmental factors on bacterial growth, such as pH, temperature, bacterial concentration, oil concentration, and incubation time. A procedure similar to that described by Liu et al. (2017) was followed. To prepare the bacterial culture, one milliliter of the best isolates that showed growth in high oil concentration was used as the zero point and introduced into 50 mL of mineral salts medium (MSM) supplemented with 5% oil, and then incubated at 35°C for about 24 h. This experiment was designed to evaluate the influence of differing pH levels (5.0, 7.0, and 8.0); bacterial concentrations (1, 2, 3 V/V); oil concentrations (100, 300, 500 mg/L) as well as temperatures (25°C, 35°C, and 45°C) on bacterial growth. To maintain the desired pH levels within the medium, appropriate adjustments were made prior to the sterilization step using 0.5 M HCl and 0.5 M NaOH solutions for pH values of 6.0 and 8.0, respectively. The microbial growth was quantified using a Hitachi U-2910 Spectrophotometer (Tokyo, Japan), measuring absorbance at a wavelength of 600 nm. In addition, the bioremediation rate was calculated according to oil residuals from the oil and grease test (American Public Health Association (APHA), 2017). All experiments were conducted in triplicate, and the resulting data were averaged for analysis.


$$B\,i\,o\,r\,e\,m\,e\,d\,i\,a\,t\,i\,o\,n\,e\,f\,f\,e\,c\,i\,e\,n\,c\,y\,\left(B\,E\%\right)=C\,i\,\_\,C\,e/C\,i^{*}\,100$$

#### 2.7.2 Influence of various concentrations of AL-MNPs on the growth of the selected bacterial strains

To evaluate the impact of AL-MNPs on bacterial activity and their potential, varying amounts of AL-MNPs (e.g., 0.01, 0.02, 0.03, 0.04, 0.05, and 0.06 g) were dissolved in 90 mL of mineral salt medium under optimal conditions of pH 7 and a temperature of 35°C. Each flask containing 90 mL of MSM with the specified quantity of AL-MNPs was supplemented with 5% vegetable oil, and then autoclaved at 121°C for 20 min. Subsequently, each flask was gently inoculated with 1 mL of a selected bacterial strain and incubated in a shaking incubator (150 rpm) at 35°C for varying intervals ranging from 3 to 9 days (Ehis-Eriakha and Balogun, 2020). Bacterial growth was finally assessed using optical spectroscopy (Akinduti et al., 2019).

#### 2.7.3 Scale-up technique

For scaling up the application, the combined use of bacterial isolates with AL-MNPs was carried out under optimized environmental conditions, including pH 7, temperature 35°C, appropriate inoculum volume, and initial oil concentrations, to maximize and accelerate the bioremediation process in the oil—water system. Likewise, the application has been conducted with two types of oil: vegetable and engine oil. The experiment was designed to evaluate the effects of treatments and time on the various concentrations (100, 300, and 500 mg/L) of both engine and vegetable oil, which were independently assessed using a two-way ANOVA. The three treatments were represented as follows: MNPs alone as a group (1), bacteria alone as a group (2), and MNPs + Bacteria as a group (3), with a non-treated group serving as the control. The impact of the incubation period was also assessed as an environmental factor, varying from 0-day (control), 3-, 6-, and 9-days. Oil and grease analysis was used to track oil residues. Tukey’s *post-hoc* test was used for pairwise comparisons after a two-way analysis of variance (ANOVA) was applied to analyze all the data. The statistical significance threshold was set at *P* < 0.05, and the results are displayed as mean ± SD. Three duplicates of each experiment were conducted. To assess the collected data, the Statistical Package for the Social Sciences (SPSS version 19. IBM Corporation) was utilized.

### 2.8 Oil degradation measurement using gas chromatography-mass spectrometry

Based on the findings of oil and grease analysis, the chemical composition of samples specifically treated with MNPs + Bacteria groups for engine oil and vegetable oil was studied, using Gas Chromatography-Mass Spectrometry (GC-MS) to analyze the oil degradation process. A Trace GC1310-ISQ mass spectrometer (Thermo Scientific, Austin, TX, United States) fitted with a direct capillary column TG–5MS (30 m × 0.25 mm × 0.25 μm film thickness) was used for analysis following three days of incubation. The temperature of the column oven began at 35°C, rose by 3°C/min to 200°C (held for 3 min), and then increased again to 280°C at 3°C/min with a 10-min pause. The temperatures of the MS transfer line and injector were kept at 260°C and 250°C, respectively. The carrier gas was helium, which flowed at a steady 1 mL/min. An Autosampler AS1300 in split mode was used to automatically inject 1 μL of diluted samples after a 3-min solvent delay. Electron Ionization (EI) mass spectra were obtained at 70 eV ionization voltages, scanning from m/z 40 to 1,000 in full scan mode, with the ion source temperature set at 200°C. Compound identification was achieved by comparing retention times and mass spectra with those in the WILEY 09 and NIST 11 mass spectral databases.

This analytical approach was used to provide detailed insights into the degradation process of engine oil and vegetable oil by assessing changes in their chemical profiles following treatment with a mix of MNPs and bacteria, facilitating a comprehensive understanding of the bioremediation process.

### 2.9 Recycling and performance assessment of AL-MNPs for oil remediation

The reusability of MNPs adds economic value. The use of AL-MNPs in oil remediation was evaluated through a recycling approach. To do so, the following experiment was conducted. In each cycle, 0.04 g of AL-MNPs were used in batch adsorption experiments at 300 mg/L of oil (representing engine and vegetable oils) for 3 days, using a magnetic bar on a rotating shaker to ensure equilibrium. Following the adsorption phase, the AL-MNPs were dehydrated and then submerged in 50 mL of 0.1 M HCl. The mixture was shaken at ambient temperature to desorb the adsorbed oil. The AL-MNPs were dried to remove excess solvents. This process was repeated until adsorption deactivation was detected.

## 3 Results

### 3.1 Water quality assessment of the collected water samples

To assess the capacity of a specific bacterial species within a known community to generate oil-degrading bacteria in correlation with the environmental conditions, studies have been conducted to evaluate the water quality of sampled sites. Table 2 represents different water quality parameters for three sites, showing that S2 has a high load of oil & grease. While S3 recorded a high total bacterial count about 20 × 10^4^ CFU/mL followed by S1 then S2 with 18 × 10^2^, 17 × 10^3^, respectively.

**TABLE 2:** Water quality assessment for different sampling sites.

Water quality parameter	Unit	S1				S2				S3			
		Min.	Max.	Mean	SD.	Min.	Max.	Mean	SD.	Min.	Max.	Mean	SD.
pH	mg/L	6.9	7.1	7.0	±0.07	7.5	7.7	7.6	±0.06	7.9	8.1	8.0	±0.07
EC	mmhos/cm	0.27	0.29	0.28	±0.01	0.46	0.48	0.47	±0.01	2.05	2.11	2.08	±0.03
TDS	mg/L	170	190	180	±0.08	390	410	400	±0.08	980	1,020	1,000	±0.15
Turbidity	mg/L	3.7	4.1	3.9	±0.02	14	16	15	±0.03	195	205	200	±0.04
Ammonia	mg/L	0.10	0.12	0.11	±0.01	0.32	0.36	0.34	±0.02	17.5	18.5	18	±0.04
Oil and grease	mg/L	0.05	0.07	0.06	±0.01	165	175	170	±0.06	11.5	12.5	12	±0.03
BOD	mg/L	9.5	10.5	10	±0.05	58	62	60	±0.02	95	105	100	±0.02
Total bacterial count (TBC)	CFU/mL	15 × 10^2^	20 × 10^2^	18 × 10^2^	±0.04	16 × 10^3^	19 × 10^3^	17 × 10^3^	±0.03	18 × 10^4^	22 × 10^4^	20 × 10^4^	±0.01

### 3.2 Isolation and characterization of powerful bacterial isolates proficient in oil degradation

**FIGURE 2:**
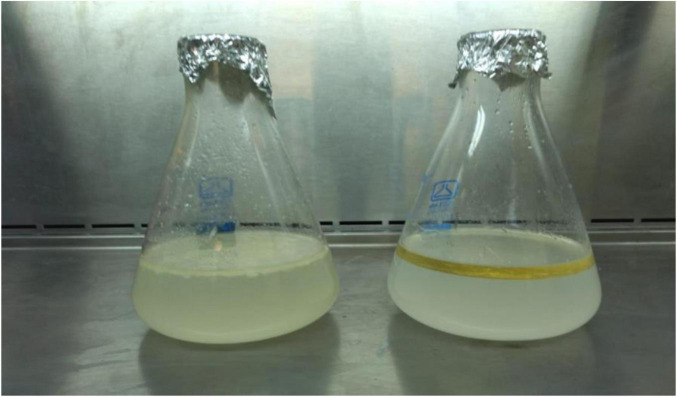
Bacterial growth selection on MSM.

**FIGURE 3:**
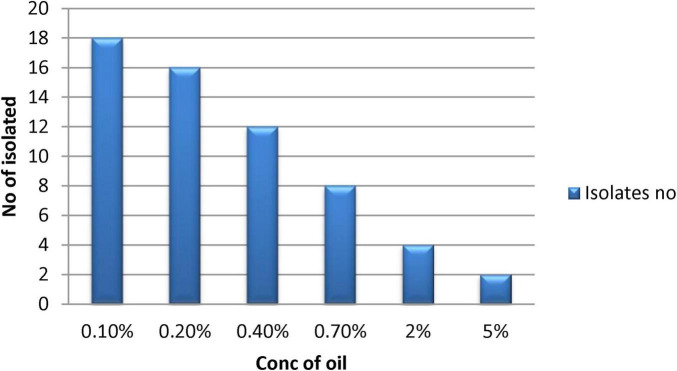
Bacterial growth against vegetable oil on MSM. The Y axis shows the percentage of vegetable oil concentration while the X axis shows the number of bacterial isolates survived.

The Mineral Salt Medium (MSM) was supplemented by 0.1% v/v of vegetable oil, which acted as the sole carbon and energy source for bacterial development. A total of 18 bacterial isolates were chosen based on their growth performance in oil presence. Ultimately, with increasing concentrations of vegetable oil, only four bacterial isolates demonstrated survival in media containing 5% v/v of oil. The survival pattern of these bacterial strains across different percentages of vegetable oil is depicted in Figures 2, 3. Among them, isolates B3 and B4 exhibited the highest growth and were selected for further identification.

### 3.3 Identification of best oil degrading bacteria using 16S rRNA analysis

**FIGURE 4:**
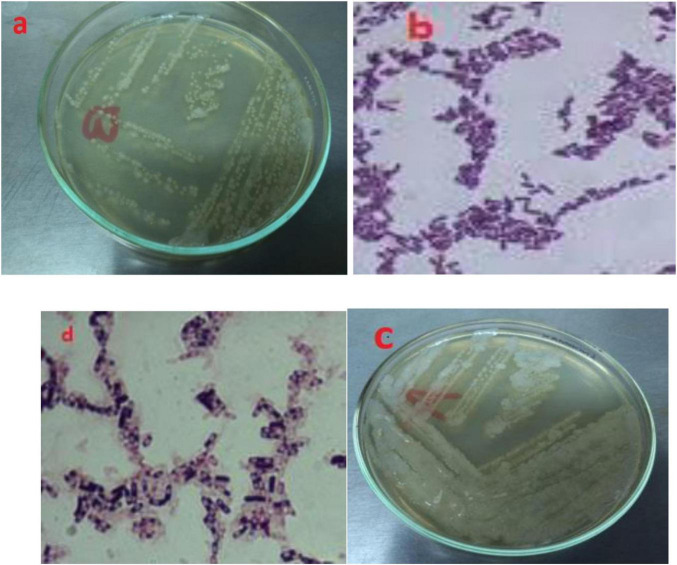
Morphology and gram staining of potential oil degrading bacteria where **(A,B)** for B3; **(C,D)** for B4.

After identification via morphology, Gram staining confirmed two Gram-positive rods bacterial isolates, B3 and B4 (Figure 4). They were 100% identical to members of the *Bacillus* genus by 500 bp 16S rRNA gene sequencing. The isolate B3 was *Bacillus flexus*, and B4 was *Bacillus pumilus*. These two species have already been reported to be oil-degrading bacteria (Ren et al., 2015). Partial 16S rRNA gene sequences of B3 and B4 isolates were lodged with GenBank and were given accession number PP939648.1 for *Bacillus flexus* (B3) and PP939649.1 for *Bacillus pumilus* (B4), respectively.

**FIGURE 5:**
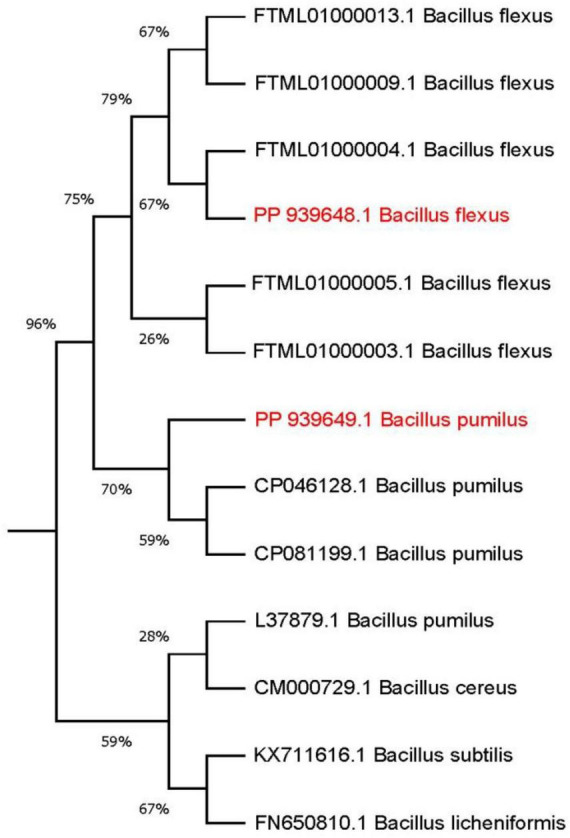
Phylogenetic tree showing the position of PP939649.1 *Bacillus pumilus* (B4) and PP939648.1 *Bacillus flexus* (B3) isolated strains. The optimal tree with the sum of branch length = 0.2065 is shown. The nucleotide sequence used for finding evolutionary relationship was ∼ 500 bp. The evolutionary distances were computed using the Maximum Composite Likelihood method and are in the units of the number of base substitutions per site.

To deepen the characterization and explore the relationship between the isolated strains and their closely related strains available on NCBI, a phylogenetic tree was constructed using the Neighbor-joining method, following the approach described by Tamura et al. (2011) and Kumar et al. (2018) (see Figure 5). Based on the observation that strain B. pumilus (B4) exhibited higher growth and OD values compared to other strains after 9 days of incubation (as shown in Table 3), this strain was selected for further optimization experiments.

**TABLE 3:** Optical density of the isolates in mineral salt broth containing vegetable oil after 9-day incubation in shaker.

Isolate	Optical density at 600 nm
B1	0.099
B2	0.050
B3	0.501
B4	1.273

Subsequently, PP939649.1 *Bacillus pumilus* strain was introduced into media enriched with vegetable oil following a 9-day incubation period, marking the initial phase of optimization studies.

### 3.4 Influence of some environmental factors on the bacterial growth

**FIGURE 6:**
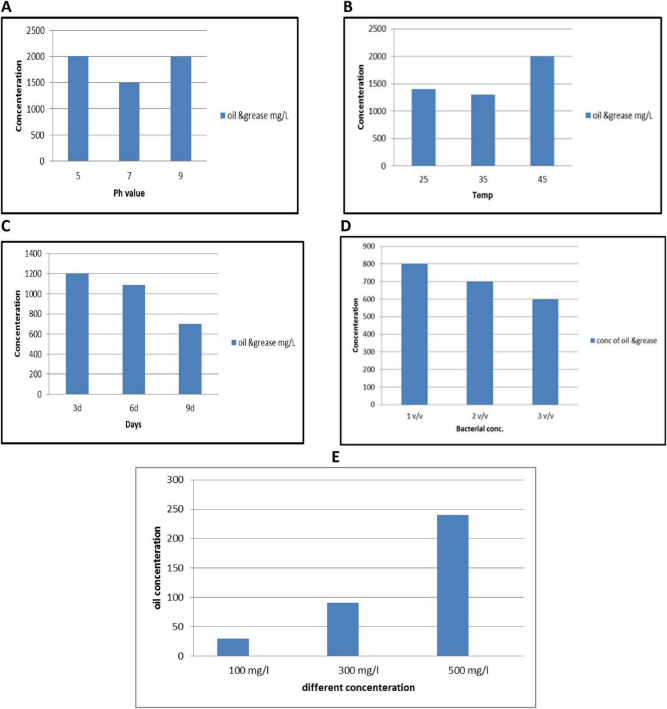
Impact of pH **(A)**, temperature **(B)**, incubation days **(C)**, bacterial inoculum **(D)**, and **(E)** initial oil concentration on the degradation oil efficiency using PP939649.1 *Bacillus pumilus*. Error bars represent standard deviation.

The growth of isolated bacterial strains was systematically assessed under varying environmental conditions, including medium pH levels (e.g., 5, 7, and 8), temperatures (e.g., 25, 35, 45°C), incubation time (3d, 6d, 9d) bacterial inoculum (e.g., 1, 2, 3 v/v) and initial oil concentration (e.g., 100, 300, 500 mg/L) as represented in Figure 6. Optimal bacterial growth was observed at a pH of 7.0 after incubation 9 d with bacterial inoculum 3v/v and at temp 35°C.

### 3.5 Characterization of produced AL-MNPs

#### 3.5.1 UV-visible characterization

**FIGURE 7:**
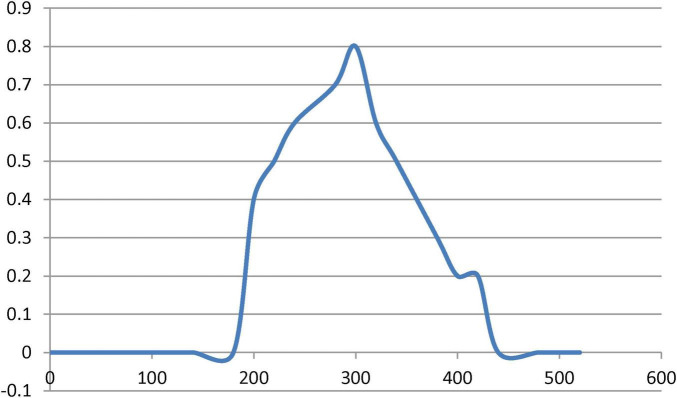
UV/Vis absorption spectra of the synthesized MNPs using the GS.

One of the most conventional techniques, UV–Vis spectrophotometer analysis, usually proceeds by assessing the change in color of the solution; consequently, absorbance confirms the structural properties of MNPs. The formation of new absorption bands confirms the formation of NPs. In Figure 7, the analysis of UV–Vis spectra demonstrated that the presence of secondary metabolites favored the stable synthesis of MNPs. *Artemisia* leaf extract can act as both the reducing agent and the stabilizer. Freshly prepared magnetite suspensions had a broad surface plasmon resonance absorption peak at around 300 nm in the UV region.

#### 3.5.2 XRD analysis

**FIGURE 8:**
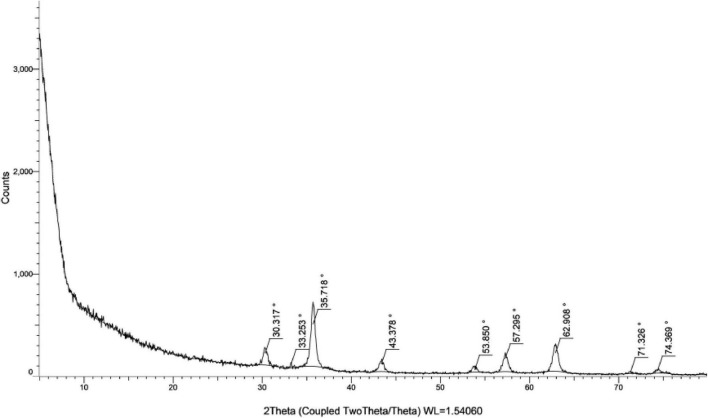
XRD patterns spectra for green biosynthesis of magnetite from GS extract.

Nine peaks appeared at 2θ = 30.317, 33.253, 35.718, 43.378, 53.850, 57.296, 62.908, 63.10, 71.326 and 74.369 correspond to the hkl planes of (201), (220), (311), (222), (400), (422), (103), (511), (111), and (520), respectively (Figure 8). These diffraction peaks correspond to the pure cubic phase of magnetite by the standard data from the Joint Committee on Powder Diffraction Standards-JCPDS file, PDF No. 19–0629-as He et al. (2017). Furthermore, there are no traceable impurities from the XRD to confirm that synthesized MNPs exhibit a well-defined, highly pure crystalline structure. These peaks of diffraction give an indication of the typical size of crystallites of the nanoparticles. The size of MNPs was determined by Scherrer’s formula as expressed in Equation (2):


D=k⁢λ/β⁢C⁢O⁢S⁢θ

β is the FWHM of the peak (He et al., 2017). From the Scherrer equation, the average crystallite size for the MNPs was in the range 20–31 nm (see Table 4).

**TABLE 4:** Calculation of MNPS size using the Scherrer equation.

Peak number	Pos. (2θ)	FWHM	Size (nm)
1	30.317	0.3825	20.01
2	33.253	0.3825	21.44
3	35.718	0.5510	24.22
4	43.378	0.4723	22.04
5	53.850	0.5723	26.37
6	57.296	0.6723	30.23
7	62.908	0.4815	29.87
8	63.10	0.6430	28.23
9	71.326	0.5654	27.18
10	74.369	0.6723	31.23

#### 3.5.3 SEM and EDX analysis

**FIGURE 9:**
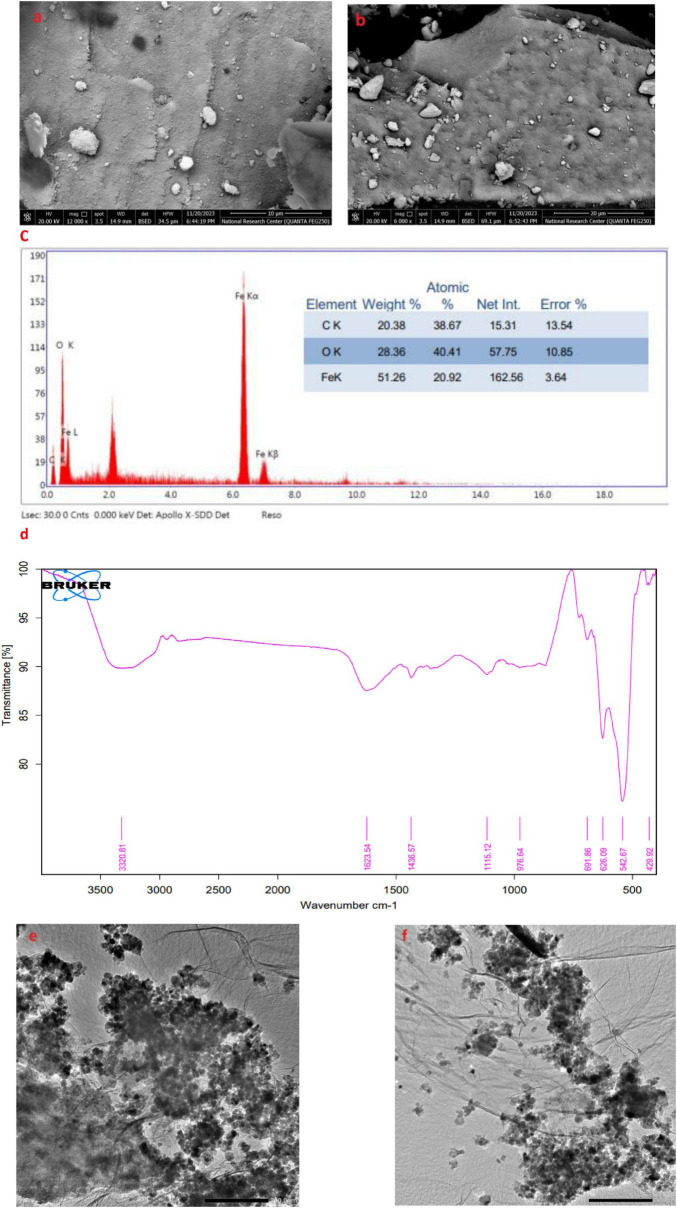
Morphological characterization of phytofabricated AL-MNPs. **(a,b)** SEM images with different magnifications (10 and 20 μm); **(c)** EDX analysis; **(d)** FTIR analysis; **(e,f)** TEM images.

It was noted that, in Figures 9a,b at 10–20 μm magnification, were a somewhat irregular spherical shape, forming aggregates in a three-dimensional arrangement. The average size of the produced nanoparticles was measured to be around 30.43 nm, indicating that the particle size fell within this range. EDEX spectrum of synthesized nano-materials were represented in Figure 9c.

#### 3.5.4 FTIR characterization

FTIR spectroscopy was applied to identify major functional groups of the *Artemisia* leaf extract and its possible role for the reduction, stabilization, and formation of MNPs. In the FTIR spectrum of aqueous extract *Artemisia* leaf and synthesized MNPs are presented in Figure 9d, the following characteristic absorption bands were noticed 3,320.81, 1,623.54, 1,115.12, 976.64, 691.86, 626.09, and 429.92 cm^–1^.

#### 3.5.5 TEM analysis

One drop of the green-synthesized MNPs derived from aqueous extract *Artemisia* leaf was placed on a copper grid for transmission electron microscopy (TEM). After the sample dried, the grid was examined using TEM at 100 kV. Figures 9e,f presents the resulting TEM image.

### 3.6 Influence of AL-MNPs on bacterial culture growth and isolation of potential MNPs bacterial isolates

**FIGURE 10:**
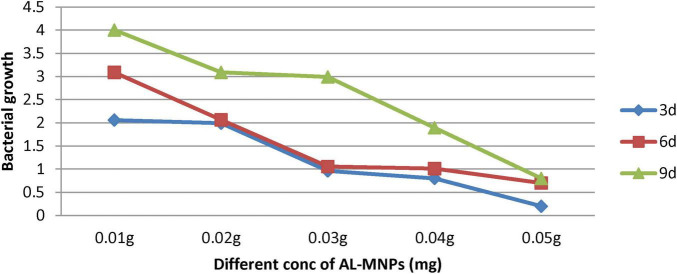
Bacterial growth at wavelength 600 nm using different concentrations of AL-MNPs (mg) at different time intervals.

The impact of AL-MNPs concentrations ranging from 10 to 60 mg on bacterial growth is illustrated in Figure 10. Notably, lower concentrations (e.g., < 40 mg) of AL-MNPs demonstrated an enhancing effect on bacterial growth over various time intervals (e.g., from 3 to 9 days).

### 3.7 Effectiveness of treatments

Table 4 shows that oil removal efficiency by MNPs, bacteria, and their combination decreased over time for both engine and vegetable oils across all concentrations (100, 300, and 500 mg/L). The combination of bacteria and MNPs generally showed the highest initial removal rates, especially over time. This consistent decline highlights reduced treatment performance with extended exposure. Our results showed that AL-MNPs alone could remove about 59% of oil at a concentration of 300 mg/L within 3 days under magnetic separation. This finding is in good agreement with the previous work, where magnetite nanoparticles showed excellent oil removal efficiencies of up to 70% for the lower chain alkanes (C9–C22) after only 72 h of treatment (Mirshahghassemi et al., 2019). Such rapid initial removal indicates a high affinity of nanoparticles for lower chain hydrocarbons, likely driven by surface properties and hydrophobic effects of sorption. Two-Way ANOVA for Comparison of the effects of experimental treatments and time, on varies concentrations of examined oil were conducted and the result is presented in Tables 5, 6.

**TABLE 5:** Results of oil removal % by three groups of treatment at different time intervals.

Concentrations	Time	Engine oil				Vegetative oil			
		(Control)	MNPs	Bacteria	Bacteria + NPs	(Control)	MNPs	Bacteria	Bacteria + NPs
100 mg/L	3d	100	41 ± 0.58^c^	95 ± 0.58^c^	10 ± 0.77^c^	100	37 ± 0.33^c^	90 ± 0.33^c^	9 ± 0.14^c^
	6d	100	39 ± 1.68^b^	20 ± 0.58^a^	10 ± 0.46^b^	100	33 ± 0.43^b^	19 ± 0.46^b^	9 ± 0.22^b^
	9d	100	39 ± 1.37^a^	18 ± 0.58^a^	10 ± 0.45^a^	100	28 ± 0.61^a^	17 ± 0.91^a^	9 ± 0.34^a^
300 mg/L	3d	300	120 ± 0.71^c^	280 ± 0.58^c^	28 ± 0.44^c^	300	100 ± 0.04^c^	250 ± 0.77^c^	26 ± 0.77^c^
	6d	300	118 ± 0.32^b^	80 ± 0.58^b^	27 ± 0.11^b^	300	95 ± 0.55^b^	70 ± 0.88^b^	25 ± 0.46^b^
	9d	300	117 ± 0.08^a^	60 ± 0.42^a^	27 ± 0.58^a^	300	90 ± 0.40^a^	55 ± 0.07^a^	25 ± 0.74^a^
500 mg/L	3d	500	400 ± 0.11^c^	500 ± 0.22^c^	360 ± 0.58^c^	500	370 ± 0.99^c^	500 ± 0.55^c^	320 ± 0.18^c^
	6d	500	380 ± 0.52^b^	400 ± 0.35^b^	290 ± 0.58^b^	500	350 ± 0.14^b^	370 ± 0.42^b^	260 ± 0.22^b^
	9d	500	370 ± 0.24^a^	380 ± 0.07^a^	280 ± 0.58^a^	500	340 ± 0.22^a^	350 ± 0.11^a^	250 ± 0.67^a^

Results (mean ± SD); A one-way ANOVA test and then Tukey’s *post-hoc* test was conducted at *p* < 0.05. Different letters (a–c) are used to indicate statistically significant differences between groups based on Tukey’s post hoc test (*p* < 0.05).

**TABLE 6:** Two ways—ANOVA analysis.

Source	DF	Seq SS	Adj SS	Adj MS	*F*-value	*P*-value
Treatment group	9	37,382	37,382	6,382	109.15	0.010
Time (days)	9	36,569	36,569	62,264	1034.62	0.020
Error	170	8,567	8,567	70	–	–
Total	189	4,20,010	–	–	–	–

### 3.8 GC-MS-technique

**FIGURE 11:**
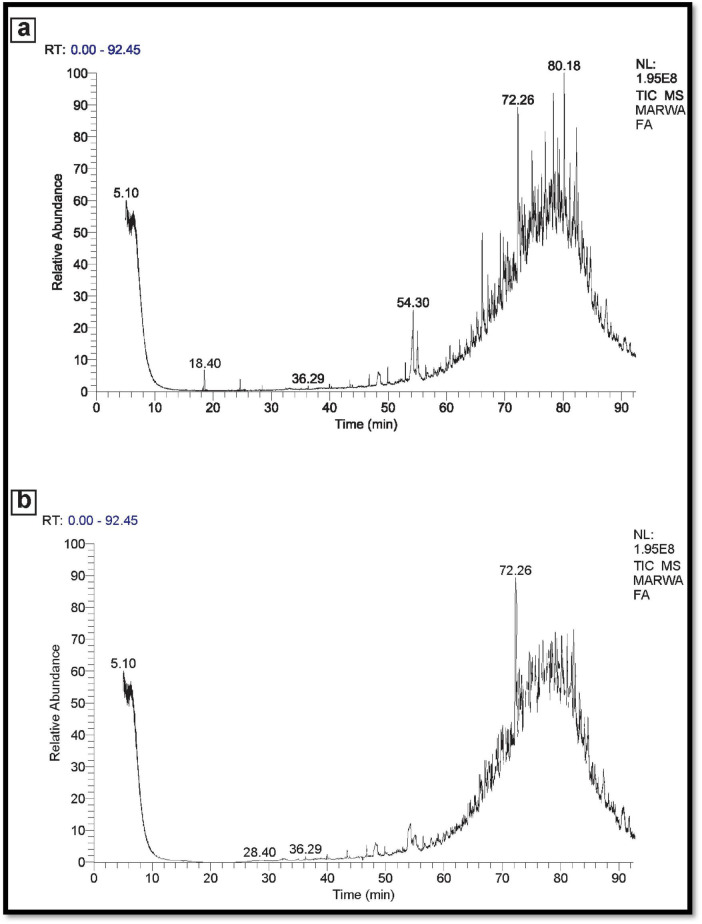
**(A)** GC-MS spectra Control used Engine oil (No inoculation). **(B)** GC-MS spectra used engine oil sample (inoculated).

**FIGURE 12:**
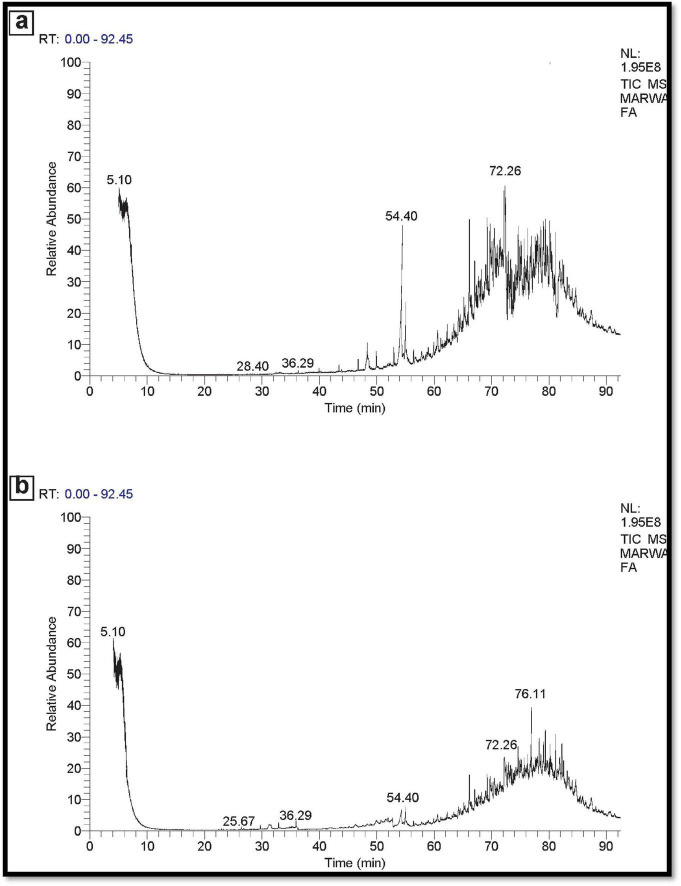
**(A)** GC-MS spectra Control used vegetable oil (No inoculation). **(B)** GC-MS spectra used vegetable oil sample (inoculated).

In the instance of used engine oil, the control set comprised 66 components, while the treated set contained 38 components. This suggests that the potential MNPs bacteria demonstrate a strong capability to degrade and eliminate 28 components of the used engine oil (Figure 11). Similarly, for used vegetable oil, the control set consisted of 50 components, whereas the treated set exhibited 30 components. This underscores the ability of the potential MNPs bacteria to degrade and eliminate 20 components of the used vegetable oil as represented in Figure 12.

Overall, these results suggest that the Nano-bioremediation approach involving the synergistic interaction between indigenous microorganisms and nanoparticles offers an affordable, environmentally, and beneficial solution for oil elimination. Where analyses possesses showing a greater ability to degrade used engine oil and vegetable oil compared to control oil sample.

### 3.9 The bioremediation mechanism of crude oil

The synergistic effect of magnetite nanoparticles (AL-MNPs) with oil-degrading microorganisms is an effective method for efficient oil bioremediation. AL-MNPs, being of enormous surface area, dispersive, and catalytically active in nature, augment bioavailability of hydrophobic hydrocarbons to a very great extent. By emulsification enhancement of the oil droplets and by augmentation of the oil—water interface, the nanoparticles make the hydrocarbons more vulnerable to invasion by microorganisms. Apart from this, the adsorptive nature of AL-MNPs helps them adsorb with toxic fractions of hydrocarbons and reduce their inhibitory effect on enzyme activity and microbial growth.

**FIGURE 13:**
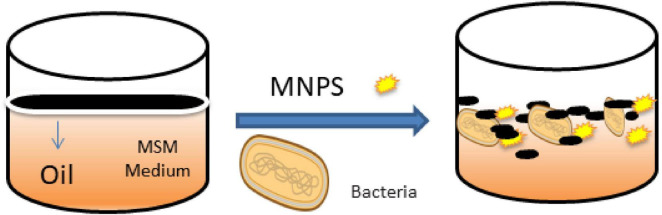
Overview of oil degradation.

Since there is better bioavailability of the oil, oil-degrading bacterial processes are induced as represented in Figure 13. Bacteria that degrade oil possess unique enzymes such as alkane monooxygenases and dioxygenases, which they use in order to catalyze degradation of hydrocarbon (Parthipan et al., 2022). Oxidation occurs at the terminal position of aliphatic hydrocarbons to yield primary alcohols, which upon secondary oxidation result in aldehydes and further fatty acids. These latter fatty acids are channeled into the β-oxidation process and are broken down into the tricarboxylic acid (TCA) cycle (Li et al., 2020). As an illustration, oxidation of a straight-chain alkane may be represented as below (Bestawy et al., 2020):


$$R-C\,H_{2}-C\,H_{3}+O_{2}+N\,A\,D\,H+H^{+}\to R-C\,H_{2}-C\,H_{2}\,O\,H+N\,A\,D^{+}+H_{2}\,O.$$

Aromatic hydrocarbons, naphthalene, are first ring-hydroxylated by dioxygenase enzymes to form cis-dihydrodiol intermediates, which are then cleaved and mineralized to carbon dioxide and water. Besides the direct induction of degradation, AL-MNPs also promote bacterial colonization and development of a biofilm on the oil droplet surface (Adlan et al., 2020). This kind of interaction favors the creation of a microenvironment that is favorable for efficient oil degradation and unfavorable for environmental stress conditions, i.e., salinity, pH fluctuation, or toxicity of certain fractions of the oil. Additionally, the magnetism of AL-MNPs favors easy recovery after treatment, thus making the nanoparticles reusable and the process cost-effective and eco-friendly (Alabresm et al., 2018).

In the current research, use of AL-MNPs in combination with oil-degrading bacteria showed considerably enhanced degradation efficiency compared to bacterium-based or nanoparticle-based treatments individually. Two processes are accountable for the enhanced efficiency: physical accessibility facilitation by the nanoparticles and biological degradation via bacterial enzymes. For instance, the nanoparticles are able to adsorb on the surface of polycyclic aromatic hydrocarbons (PAHs), which produces localized high hydrocarbon concentrations that initiate microbial degradation alongside with decreasing PAH toxicity (Ehmedan et al., 2021).

Combined, this micro-biology-nanotechnology mix delineates the two disciplines’ application versatility in oil bioremediation. Aside from ensuring improved treatment efficiency, the approach enjoys operational advantages of ease in recycling nanoparticles and reducing chemical pollutant load. Microbial population dynamics at the genetic level, nanoscale interactions between bacteria and nanoparticles, and scalable loading of nanoparticles for mass-volume bioremediation applications remain subject to optimization by further research (Salama et al., 2023).

### 3.10 Comparison with other adsorbents

Comparative evaluation of oil removal efficiency and adsorption capacities in various studies provides in-depth information regarding the effectiveness of magnetite nanoparticles (AL-MNPs) and oil-degrading bacteria for environmental remediation. Data in Table 7 graphically represent the in-depth synergy between AL-MNPs and oil-degrading bacteria, i.e., oil removal from aquatic environments. Synergistic application of AL-MNPs and oil-degrading bacteria resulted in 90% oil removal in 3 days when optimal conditions (pH 7.0, Temp 35°C) were applied as opposed to single treatments under which oil removal was less. The observation has been in accordance with other reported results on enhanced degradation efficiency where nanoparticles and microbes have a synergistic effect. For instance, Alabresm et al. (2018) found that magnetite nanoparticle in combination with bacteria increased the oil removal rate, i.e., how nanoparticles enable the enhancement of the bioavailability of the hydrocarbons, thereby allowing microbial degradation.

**TABLE 7:** Comparison of oil removal efficiency with AL-MNPs and oil-degrading bacteria in different studies.

Study	Treatment	Efficiency (%)	Conditions
Current study	AL-MNPs (alone)	59	Oil concentration 300 mg/L; pH 7.0; Temp 35°C; 3 days.
Current study	Oil-degrading bacteria (alone)	72–80	Bacterial inoculum 3 v/v; pH 7.0; Temp 35°C; 6 days.
Current study	AL-MNPs + oil-degrading bacteria	±90	pH 7.0; Temp 35°C; Oil concentration 300 mg/L; 3 days.
Mirshahghassemi et al. (2019)	Iron oxide nanoparticles (alone)	70	Oil concentration 150 mg/L; pH 7.0; 4 days.
Li et al. (2020)	Bacteria (alone)	50–60	pH 6.0; Temp 30°C; 7 days.
Alabresm et al. (2018)	Magnetite nanoparticles + Bacteria	80	Oil concentration 100 mg/L; pH 6.5; Temp 25°C, 3 days.

Besides, the experiment also showed that AL-MNPs alone had degraded 59% of the oil in 3 days, a remarkable finding that indicates their potential as oil degradation and adsorption pollutants. Mirshahghassemi et al. (2019) also showed that magnetite nanoparticles removed 70% of the oil in the same time, supporting the claim that magnetite nanoparticles have high affinity for oil, the higher the lower chain alkanes (C9–C22). Nevertheless, nanoparticles’ removal efficiency by itself seems to plateau with high concentrations, perhaps due to saturation of oil components and therefore the additional constraint on removal efficiency.

### 3.11 Regeneration and reusability

**FIGURE 14:**
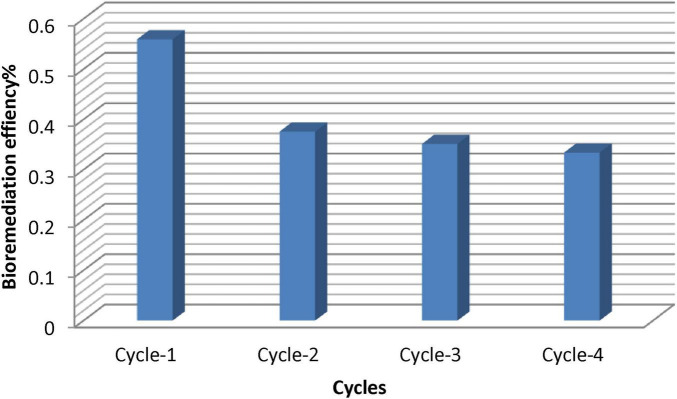
Recycling investigations for reuse of AL-MNPs.

Reusability of AL-MNPs was always in decreasing order for every cycle. During the first cycles, removal efficiency was good, but when repeated, removal efficiency decreased as represented in Figure 14. This is because active sites of the nanoparticle are gradually saturated in repeated cycles, and it becomes difficult for them to regenerate in repeated cycles. This is as noted in other studies on regeneration of adsorbents from nanoparticle-based adsorbent (Haripriyan et al., 2022). In our studies, AL-MNPs were extremely reusable with a decline in oil removal capacity after four cycles. However, even after multiple cycles, the nanoparticles retained high potential to aid in the degradation of oil, demonstrating their usability for extended bioremediation.

## 4 Discussion

Petroleum is a toxic compound and a complex organic mixture of hydrocarbons. Mass spectrometry analysis shows that it contains more than 17,000 chemical compounds, including mono- and polycyclic aromatic hydrocarbons, cyclic alkanes, and various chain alkanes. Due to the diverse chemical and physical properties of its components, their biodegradability and environmental impacts vary greatly (Zhang et al., 2019). Oil spills have been linked to chemical contamination, oxygen depletion (Moskovchenko et al., 2020), human health risk (Adeola et al., 2022), and long-term ecosystem damage (Mugendi, 2020) across the world. In this investigation, the characteristics of water samples collected from the three sites were represented, including physical, chemical and bacteriological measurements, taking into account oil and grease levels, Biological oxygen demand (BOD), and total bacterial account (TBC). At location of Al Qanatir Al Khayriyyah city, Al-Qalyubia governorate (S2), which represents surface water close to the marina, oil and grease levels and BOD reached 170 and 60 mg/L, respectively. This is obviously attributed to accidental oil contamination during ship docking. Meanwhile, TBC at the same location (S2) markedly exceeded its counterpart in the Ismalia canal at Belbies city, Al-Sharqia governorate (S1), which represents the River Nile reaching 15,000 and 1,200 CFU/mL, respectively, indicating human activity. These findings are consistent with those of Al-Samhan et al. (2017), who observed a similar trend in River Nile water quality during oil spills, noting elevated oil and grease levels, elevated BOD values, and TBC. Conversely, El-Khadrawia, Menofia Governorate (S3), representing wastewater, displayed severe contamination with elevated turbidity, ammonia, BOD, and TBC, while showing a notably lower oil and grease concentration of 12 mg/L compared to Al Qanatir Al Khayriyyah site (2). Suggesting that the bacterial count may be responsible for the low oil and grease levels at the contaminated location. Therefore, the following step was to study the bacterial strain that suppresses oil levels in the aquatic environment (Ghiloufi et al., 2019).

The concept of bioremediation stands out as a dependable and eco-friendly method for eliminating oil pollutants from various environments, with microorganisms, particularly bacteria, playing a pivotal role in this process. Previous Research has unveiled diverse groups of oil-degrading bacteria and their potential has been extensively studied across diverse marine environments, including water bodies and sediments (Venkateswaran et al., 1991). Furthermore, other research has documented bacterial adaptation to varying concentrations of crude oil (Sharma and Schiewer, 2016). In the study by Xiao et al. (2024) investigated the selection of a useful consortium for remediating oil-contaminated soil was explored shedding light on the dynamic interplay between bacterial communities and crude oil concentrations.

Genus *Bacillus* species are often found in oil-contaminated environments due to their ability to thrive in low-nutrient conditions (Macaulay and Rees, 2014). *Bacillus* species are known for their bioremediation capabilities, with reports of marine oil contaminants being mitigated by these bacteria (Chikere et al., 2009). These findings collectively indicate that *Bacillus* species exhibit high adaptation to diverse and harsh environments. Various *Bacillus* species have been extensively studied for their potential in oil bioremediation or their close association with oil in different contaminated areas (Wald et al., 2015). In our study it’s worth noting that *Bacillus* species exhibit a higher degradation potential for used engine oil (68%) compared to crude oil (84%) as reported by Raju et al. (2017). Borah and Yadav (2017) also approved the ability of *Bacillus* species to biodegrade various hydrocarbons, with percentages of 42% for used engine oil, 57% for crude oil, 67% for diesel oil, and 72% for kerosene.

Our findings support previous studies that highlighting the importance of maintaining pH within the range of 5–8 and temperatures between 30 and 40°C for effective bacterial bioremediation of hydrocarbons and thus align with existing literature (López and Antuch, 2020; Borah and Yadav, 2017). These conditions facilitate enhanced bacterial activities and the production of degradative ability, crucial for efficient hydrocarbon bioremediation. Furthermore, the formation of biofilms in hydrocarbon-contaminated environments may promote microbial adaptation to the limited bioavailability of hydrophobic compounds. This adaptation includes the up regulation of genes encoding hydrocarbon degradative enzymes, which play a pivotal role in microbial remediation of hydrocarbon-polluted environments.

Regarding nanoparticles synthesis, it is evidenced from the clear color transformation represent MNPs formation. Moreover, previously in literature, evidence was there that presented a similar trend over the UV range concerning MNP using plant extracts (López and Antuch, 2020). Our findings are in agreement with reports of the absorbance peak for magnetite synthesized using different plant extracts at around 290–360 nm (Priya et al., 2021). The XRD spectrum shows ten characteristic peaks that confirm the formation of the nanoparticles represented in Figure 8. The image illustrates that the MNPs have nearly uniform crystalline structures with most of the particles highly cubic in shape. Their sizes are in the same order or slightly higher than that calculated from XRD studies. Hence, the XRD technique is efficient in estimating the average crystallite size while the TEM technique is suitable for measuring particle size (Tamiji and Nezamzadeh-Ejhieh, 2019). Confirmation of the existence, elemental composition, shape, and size of the phytofabricated MNPs was performed by SEM images at two different magnifications of 10 and 20 μm along with EDX analyses as represented from Figures 9a–c. As MNPs appear in these results are in agreement with earlier reports on MNPs prepared using different plant extracts, where the particles were also seen to be hexagonal in shape and with no defects on their smooth surfaces (Suter et al., 2021). Another report indicated that MNPs prepared with Justicia adhatoda leaf extract had agglomerated cubic-shaped structures. The different precursors and capping agents (plant extracts) used can be the cause of variations in the morphology and shape of the phytofabricated NPs (Beura et al., 2021). Sharp signals for iron and oxygen can be seen in the EDX spectrum of the MNPs reflected in Figure 9c, suggesting that the NPs are nearly impurity-free. According to elemental analysis, the produced NPs are of high purity and quality because they include 20.92% iron and 41.51% oxygen. The EDX spectrum showed 38.67% carbon, most likely as a result of the plant extract’s bioactive ingredients (Basavegowda et al., 2017). The quantity of oxygen present would suggest that the plant’s phytochemical groups contributed to the reduction of iron ions and to the stabilization and capping of the resulting MNPs (Gupta et al., 2018).

FTIR results confirm the presence of certain bands that appeared as follow; the broad band in 3,320.81 cm^–1^ represented O-H stretching for alcohol and phenolic groups, which may be linked with carboxylic acid groups. Absorption peaks at 1,623.54 and 1,115.12 cm^–1^ were due to the presence of C-H bonds in aliphatic hydrocarbons of terpenoids. Other peaks, at 976.64 cm^–1^ and 691.86 cm^–1^ were for C = O stretching vibrations of carboxylic acids, C = C bonds in olefinic group of aromatic terpenoids, C–H bending vibration of aliphatic hydrocarbons and C–O bonds of phenolic compounds, respectively. The peak at 626.09 cm^–1^ was assigned to C–H bending vibrations of para-disubstituted aromatic protons. Two extra bands were recorded at 542.67 cm^–1^ due to the bending vibration of ortho-disubstituted aromatic protons (Tümen et al., 2018). The Fe–O stretching characteristic band was below 500 cm^–1^ with weak bands at and 429.92 cm^–1^ which confirm the presence of magnetite NPs, in accordance with previous reports by Roy et al. (2017). The presence of phenolics, flavonoids, terpenoids, and tannins was evidenced in the current FTIR and also reported by literature Saratale et al. (2021). Hydroxyl groups of phenols and flavonoids are necessary for the formation of NP through the reduction of metal ions, while terpenoids may help in the oxidation of aldehydes to carboxylic acids and further enhance the reduction of metal ions. However, the two are also thought to participate in the reduction of metal ions into stabilized NPs through active chelation as evidenced from the carbonyl peaks. This reduction is hypothesized to occur through the release of reactive hydrogen atoms from the enol to the keto form during the tautomeric transformations of flavonoids (De Marco et al., 2022). Various studies have highlighted the potential for nanoparticles (NPs) to exert synergistic or antagonistic effects on microbial growth. NPs can physically damage bacterial cell walls or induce oxidative stress, leading to the generation of reactive oxygen species (ROS) and conferring antibacterial properties. Conversely, NPs may inhibit bacterial growth due to their high specific surface area and electron release capabilities. The generated electrons can enhance enzymatic activity in the bacterial membrane, accelerating electron transport chain processes and aiding in bacterial metabolism (Rajput et al., 2018).

In our study the increase in bacterial cell density with lower AL-MNPs concentrations suggests microbial adaptation to their presence. However, concentrations exceeding 40 mg inhibited bacterial growth, likely due to stress induced by AL-MNPs overload and agglomeration, leading to toxic effects on the bacterial community. The inhibition of bacterial growth at higher AL-MNPs concentrations may be attributed to the production of ROS, such as O^2–^, OH^–^, and O_2_, by the MNPs, resulting in oxidative stress and subsequent damage to cellular components (Sharma et al., 2019). According to Bhatia and Nath (2022), the lag phase of microbial development may be reduced, while exponential and stationary phases may be prolonged by the presence of NPs. Kiran et al. (2016) observed that Nocardiopsis MSA13A exhibited faster growth in the presence of magnetite nanoparticles. This might be due to the microbial energy acquisition by the oxidation of ferrous (Fe^2+^) to ferric (Fe^3+^) ions.

Furthermore, AL-MNPs enhance the bioavailability of hydrophobic compounds as bioavailability by producing biosurfactants through microorganisms, thus supporting the bioremediation process (Cameotra et al., 2010). Most of the NPs have iron composition; biosurfactant synthesis is required in bacteria which would serve as enzyme activator helping bacteria and resulting in microbial development supporting the bioremediation process (Kameswaran et al., 2021).

Our study focused on the efficiency of magnetite nanoparticles and oil-degrading bacteria on two types of oil removal. These results will be highly valued in further understanding the nano-bioremediation process and its possible impact on aquatic ecosystems. Interesting in our results was the limited removal efficiency of oil-degrading bacteria in the early stages of treatment-a negligible oil degradation recorded within 72 h increased to about 80% within 6 days. This agrees with data obtained from a similar study done by Alabresm et al. (2018), where initially, limited degradation was observed owing to low bacterial counts; considerable removal occurred after some time due to an increase in bacterial population.

Meanwhile, when the AL-MNPs are applied in a synergistic effect with oil-degrading bacteria, the removal efficiency of oil attained as high as 90% after 3 days, while bacteria’s maximum degradation after 6 days was 72–80%. Synergy between nanoparticles and bacteria is one of the critical points, since our findings have indicated that in the presence of AL-MNPs, both degradation kinetics increased and the total removed amount of oil was enlarged, enhancing the idea of nanoparticles acting merely by facilitating contact between the bacteria and compounds of the oils.

Our findings align with earlier observations that nanoparticles can become saturated with oil, limiting further removal capacity (Fossati et al., 2019). This saturation effect highlights the necessity for optimal nanoparticle concentrations to maximize efficacy in oil remediation, underscoring the importance of carefully designed experiments to explore this parameter further. Besides, the stability of nano-bioremediation under optimum conditions of pH 7.0 and 35.0°C supports the feasibility of the proposed approach under different environmental conditions. The combination of AL-MNPs with oil-degrading bacteria contributed not only to increasing the efficiency of oil removal but also may reduce the probable toxicity due to petroleum spills and serve as an environmentally friendly alternative to the classical treatments using dispersants that can result in toxic impacts on aquatic organisms. This study confirms the potential of nanoparticles in combination with microbial approaches for efficient bioremediation of petroleum hydrocarbons (Ma et al., 2022). Results from this study point toward a promising future direction, especially toward understanding the exact mechanisms of oil removal and scaling up the technology in field applications. The degradation of the engine oil and vegetative oil was represented by comparison of the gas chromatography-mass spectrometry (GC-MS) analysis of the no degraded oil (control) and the sample after incubation with potential bacterial MNPs, for each experiment. NPs applications have been suggested as successful heightened oil retrieval techniques (Agista et al., 2018). Although NPS can support microbe processes, some relevant studies have identified the NP’s impact for the biological response rates (Shin and Cha, 2008). The developed bustle of NPS is usually denoted by their exclusive characteristics and great accessible active surface areas and functionality (Yadav et al., 2022). Additionally, the combination of nanotechnology with enzymatic routes in the bioremediation activity could take the lead to overwhelming activity (Chettri et al., 2024). The magnetite nanoparticles (MNPs) are recognized for their ability to enhance bacterial growth and facilitate the cleanup of hydrocarbon-contaminated environments (Liu et al., 2017).

When compared with this, the only application of oil-degrading bacteria was 72–80% removal efficiency after 6 days, and it was lesser than for nanoparticle-mediated treatment. Comparative slower rate in removal suggested that bacteria rely on natural bioremediation process, and it would require more time to completely degrade oil pollutants if constituents of oil are hydrophobic and less bioavailable. Synergism of AL-MNPs with oil-degrading bacteria significantly enhanced the removal of oil to nearly 90% within 3 days. This is due to the fact that the AL-MNPs can promote the growth of the bacteria by providing a good surface for the bacteria to attach, increasing the exposure of the bacteria to oil molecules. The magnetic nature of the nanoparticles also facilitates separation and recycling of the nanoparticles after oil degradation, making the process economically and environmentally feasible compared to other processes that utilize more advanced removal mechanisms (Maheria et al., 2021). Moreover, AL-MNPs should be able to induce destabilization of larger oil droplets through emulsification, enhance bacteria attachment area along with other degradation. As indicated by GC-MS analysis, low-chain alkanes (C9–C22) were drastically degraded in the mixed system where bacteria utilized AL-MNPs to get the essential carbon and iron to drive their metabolism. The observation concurrs with the previous literature, (e.g., Li et al., 2020), where synergic impact of nanoparticles on efficiency in the bioremediation process by bacteria was demonstrated.

The recyclability of AL-MNPs, is a major parameter to determine their stability and eco-friendliness, particularly in the treatment of wastewater. Recyclability has multiple advantages, such as reduced operational costs, reduction in waste product generation, improvement in understanding adsorption processes, and improvement in the economic viability of the entire process (Bala et al., 2022). But although reusability is of utmost relevance, research work on regenerating nanomaterials, most importantly oil bioremediation, is relatively little, with not much more than a few few-quality studies having been conducted (Jain et al., 2022). Regeneration of nanoparticles such as AL-MNPs happens only when active sites are present on the surface of the nanoparticle following the adsorption phase (Priya et al., 2024). In the present investigation, reusability tests of AL-MNPs were carried out after the adsorption of oil in bioremediation experiments. The regeneration of AL-MNPs was performed in different cycles by a mixture of deionized water and weak acid washes, for instance, 0.2 mol/L HCl. Regeneration is possible for repeated cycles of degrading oil using reused nanoparticles.

## 5 Conclusion

Crude oil and petroleum product spills have profound impacts on water organisms. The present study explores the synergism between magnetite nanoparticles green-synthesized using plant extracts (AL-MNPs) and oil-degrading bacteria to enhance oil removal under laboratory-controlled conditions. AL-MNPs were found to enhance bacterial growth, and doses of < 40 mg promoted bacterial growth over varied time intervals of times. Synergism between AL-MNPs and some bacterial strains led to almost 90% oil removal within 3 days. GC-MS analysis testified the removal of 50% lower-chain alkanes (C9–C22) and 30% higher-chain alkanes (C23-C26). AL-MNPs were found to be recyclable with zero progressive loss of efficiency of removal of oil after four cycles and thus amenable for future bioremediation applications. Green synthesis of AL-MNPs using plant extracts and synergism with oil-degrading bacteria is an eco-friendly and cost-effective methodology compared to conventional recovery.

## Data Availability

The data presented in the study are deposited in the NCBI repository, accession numbers PP939649.1 *Bacillus pumilus* (B4) & PP939648.1 *Bacillus flexus* (B3).

## Footnotes

1
http://www.ncbi.nlm.nih.gov/blast
